# Nitrogen Deposition Enhances Photosynthesis in Moso Bamboo but Increases Susceptibility to Other Stress Factors

**DOI:** 10.3389/fpls.2017.01975

**Published:** 2017-11-16

**Authors:** Rui Zhang, Jiasheng Wu, Quan Li, Heikki Hänninen, Chunju Peng, Hang Yao, Xinzhang Song, Yeqing Ying

**Affiliations:** ^1^State Key Laboratory of Subtropical Silviculture, Zhejiang A&F University, Hangzhou, China; ^2^Tianmu Mountain Forest Ecosystem Research Station, Hangzhou, China

**Keywords:** vascular plants, forest trees, invisible injury, bioindicators, photochemistry, risk assessment

## Abstract

Atmospheric nitrogen (N) deposition can increase the susceptibility of vascular plants to other stresses, but the physiological basis of such a response remains poorly understood. This study was designed to clarify the physiological mechanisms and to evaluate bioindicators of N deposition impact on vascular plants. We evaluate multiple physiological responses to ~4 years of simulated additional N deposition (30–90 kg N ha^−1^ year^−1^) on three age-classes (1a, 3a, and 5a) of Moso bamboo. A saturating response to the additional N deposition was found both in foliar N concentration and in P_n_. However, 3- and 5-year-old bamboo seemed to be less tolerant to extremely high N deposition than 1-year-old bamboo since they were saturated at a lower N addition. Furthermore, C/N/P stoichiometric ratios were very sensitive to N deposition in all three-age classes of bamboo, but the responses to N deposition in the various age-classes were diverse. We also found that the highest additional N deposition suppressed stomatal conductance and transpiration rate, suggesting an induced water stress. The stress induced by the high N load was also seen in photochemistry, where it reduced potential and actual photosynthetic use of light energy, diminished photo-protection capacity, and increased risk of the photo-damage. High additional N deposition contributed to a decrease in the foliar soluble protein contents and to an increase in the peroxidase activity (POD). Our study suggested, for the first time, that although the photosynthetic rate was enhanced by the increased N deposition in Moso bamboo, long-term high N load causes negative effects, such as damage to photosystem II. In Moso bamboo photochemical parameters are more sensitive to N deposition than photosynthetic rate or foliar N concentration. Furthermore, plant age should be taken into account when assessing plants' susceptibility to changes in global change drivers, such as N deposition. These findings facilitate the revealing of the risks potentially caused to vascular plants by increased N deposition before any visible symptoms of injury are seen.

## Introduction

As a result of human activities, such as the use of agricultural fertilizers and fossil fuels, atmospheric nitrogen (N) deposition rate has increased globally by as much as 10 times over the past 150 years, and the rate is projected to be doubled by 2050 (Galloway et al., 2004; Meunier et al., 2016). N deposition comes in gaseous (NH_3_, NO, N_2_O, and N_2_) or dissolved (NH4^+^ and NO3^−^) form, and nitrogen from ammonium (NH4^+^) and nitrate (NO3^−^) are the dominant forms of N in the bulk deposition (Liu et al., 2013). The increased N deposition has raised concerns about its threats to ecosystem structure and function (Phoenix et al., 2012; Meunier et al., 2016). The major detrimental impacts of N deposition include soil acidification, declining biodiversity, toxic effects on sensitive species, and increased susceptibility of plants to other stresses (Phoenix et al., 2012; Valliere and Allen, 2016; Wedlich et al., 2016). Understanding those responses and the mechanisms behind them is an important step to assess the N deposition impacts and to project the environmental risks this key global change factor causes to plants and ecosystems in the future (Phoenix et al., 2012).

In order to monitor increased N deposition rates and its impacts, most studies have so far focused on physiological responses of highly sensitive species growing in the sensitive heathland and bog ecosystems, such as bryophytes and lichens, due to their absence of well-developed cuticle (Phoenix et al., 2012; Ochoa-Hueso and Manrique, 2013). Foliar chemistry, especially N concentration, is widely used as an indicator of increased N deposition (Ochoa-Hueso and Manrique, 2013). N can be absorbed across the surface area easier in bryophytes than in vascular plants, implying in the former a higher probability of increased tissue N concentration in response to the increased N deposition (Phoenix et al., 2012). Increased tissue N can be invested to increase the photosynthetic capacity (Reich et al., 1999), and therefore bryophytes can benefit from moderate doses of N deposition by growth stimulation (Phoenix et al., 2012). However, increased N deposition can cause declines in bryophyte abundance (Phoenix et al., 2012). For example, N deposition at the rate of 35 kg ha^−1^ year^−1^ caused an 84% loss of *Rhytidiadelphus squarrosus* abundance (Arróniz-Crespo et al., 2008), and *Sphagnum balticum* nearly disappeared at an N addition of 15 kg ha^−1^ year^−1^ (Granath et al., 2009b). In addition to examining the effects of N addition to plant abundance, responses of physiological traits, such as photosynthetic and photochemical parameters and enzyme activities, have been commonly used as valuable tools for assessing the risks of increased N load in the heathland and bog ecosystems (Arróniz-Crespo et al., 2008; Neves et al., 2009; Tremblay et al., 2012; Ochoa-Hueso and Manrique, 2013).

In forest ecosystems, vascular plants are not as sensitive as bryophytes to N deposition, and in most cases, no visible symptoms of injury can be found under realistic dose levels of N deposition (Binkley and Högberg, 2016). On the contrary, enhanced forest productivity caused by increased N deposition has been commonly reported as a result of a direct fertilizing effect (Pregitzer et al., 2008; Phoenix et al., 2012). Although little evidence has been found to support the idea that increased N deposition is harming forests, major concerns of increased risk of damage due to abiotic stress caused by the increased N concentration remain (Friedrich et al., 2012; Valliere and Allen, 2016). The increased N deposition can also increase the N:P ratio in plants, leading in this way to nutrient imbalances despite of the enhanced growth (Phoenix et al., 2004; Binkley and Högberg, 2016; Song et al., 2016a). However, so far relatively few studies have examined the physiological responses of tall-growing species, such as trees in forest ecosystems, to the increased N deposition. Furthermore, studies addressing the physiological mechanisms of the N responses in the tall-growing plants are also scarce. This is unfortunate, because understanding these mechanisms is crucial for understanding and monitoring the stress induced by N deposition before any visible symptoms of injury are seen in the trees.

Moso bamboo (*Phyllostachys edulis*), a woody fast-growing species of Gramineae family, forms a total of 4.43 million ha of plantation forests in subtropical China (SFAPRC, 2014). It has a high growth rate and it can reach a mean height of 10–20 m (Peng et al., 2013; Song et al., 2016b). Moso bamboo forests not only provide textile and structural products (Shao et al., 2010; Dixon and Gibson, 2014), but they also serve as an important carbon sink in China (Du et al., 2010; Song et al., 2011). Currently, bamboo is subjected to an increasing N deposition. In Southeastern China, the major bamboo-growing region, annual bulk deposition rate reached 24.2 kg N ha^−1^ in the 2000s, with an average rate of increase of 0.56 kg N ha^−1^ year^−1^ (Liu et al., 2013). N deposition has been shown to accelerate decomposition of leaf (Song et al., 2015) and fine root litter (Song et al., 2017), increase leaf N content and N:P ratio (Song et al., 2016a), and decrease soil microbial diversity (Li et al., 2016) in Moso bamboo plantations. However, few studies have evaluated the physiological mechanisms of bamboo response to the increasing N deposition, or assessed the risks the increased N deposition may cause to this species, which is important for the sustainability of the ecosystem services of large areas in subtropical China.

The objectives of this study were (1) to evaluate the physiological mechanisms of the response to different levels of N deposition in Moso bamboo; (2) to examine if extremely high N deposition causes detrimental effects on bamboo growth, or if it induces physiological stress; and (3) to evaluate potential bioindicators of N load. We investigated how photosynthetic parameters, photochemistry, enzyme activities, and foliar chemistry in trees of three age-classes of bamboo respond to three levels of additional N deposition. We hypothesized that: (1) photosynthetic activity in Moso bamboo is sensitive to N deposition, with moderate N addition increasing and further N addition decreasing the rate of net photosynthesis; (2) changes in the photosynthetic activity are associated with foliar chemistry, especially N concentration of the leaves; and (3) extremely high N deposition induces stress, which is seen in several physiological parameters of the leaves. Testing these hypotheses will provide novel results improving the understanding of the physiological mechanisms of Moso bamboo response to the increased N deposition and the evaluation of the risks the increased deposition may cause before any visible symptoms of injury are seen in the Moso bamboo trees.

## Materials and methods

### Study site

The study site is located in Lin'an, Zhejiang Province, in southeastern China (30°14′ N, 119°42′ E, 157 m altitude). It has a subtropical, monsoonal climate, and clear-cut seasons. Mean annual precipitation and temperature are 1,420 mm and 15.6°C, respectively. Observed maximum and minimum temperatures are 41.7 and −13.3°C, respectively. The landscape is hilly and altitude ranges from 100 to 300 m.

The Moso bamboo plantations examined in the present study were established in 2001. They were managed using routine practices including weed control and annual application with compound fertilizer (N: P_2_O_5_: K_2_O, 15: 6: 20, 450 kg ha^−1^) in September. The dominant underground species is *Viola prionantha*. The soil type is acidic. As a result of long-term management activities, Moso bamboo plantations are characterized by alternating high- and low-recruitment years (Song et al., 2016a). The recruitment of Moso bamboo shoots at the present study site was high during even-numbered years (e.g., 2012, 2014, 2016) and low during odd-numbered years (e.g., 2011, 2013, 2015). Moso bamboos of more than 4 years old are usually harvested in November during even-numbered years to maximize the economic benefits. Thus, the Moso bamboo plantations are uneven-aged forests with a 2-year difference in the age of the trees (Song et al., 2016a). The characteristics of the study site at the beginning of the experiment are summarized by Song et al. (2015).

### Experimental design

Experiments were arranged in a complete random design with 12 treatments by three replicates. Moso bamboo trees of three age classes in the plantation stand were used in this study. At the beginning of the experiment the trees were 1-, 3-, or 5-year old. Here, we use their initial age (1a, 3a, and 5a) to represent these trees, even though they were at the end of the experiment 3 years older. For each age class of Moso bamboo, four levels of N addition were applied: Ambient deposition with no addition (N0, control), low addition (NL = 30 kg N ha^−1^ year^−1^), medium addition (NM = 60 kg N ha^−1^ year^−1^), and high addition (NH = 90 kg N ha^−1^ year^−1^). These rates of N addition were determined on the basis of the current local N deposition rate of 30.5 kg N ha^−1^ year^−1^ (Xie et al., 2008; Song et al., 2017). Each replication was conducted in a 20 × 20 m plot, which was separated by a 20-m-wide buffer area. Ammonium nitrate (NH_4_NO_3_) was used as N source, because it was closest to the chemical composition of the local N deposition (NH4^+^ and NO3^−^ accounting for 56.1 and 43.9%, respectively) (Song et al., 2017). From January 2013 to July 2016, the N addition treatments were carried out at the beginning of each month. Quantified NH_4_NO_3_ was weighed according to the N addition rate and dissolved in water. NH_4_NO_3_ solution was sprayed evenly onto the forest floor of each plot. Each control site received an equal amount of N-free water (Song et al., 2015).

In the early August 2016, 9 Moso bamboos (three for each replicate) were selected for each treatment. A 10 m-height-scaffold was established around each sample bamboo. In each of the sample bamboos, three mature and healthy leaves on the southern side at the mid-upper canopy were selected for photosynthetic and fluorescence measurements.

### *In situ* measurements of gas exchange, fluorescence, and chlorophyll content

Gas exchange measurements were carried out with Li-6400 (Li-Cor, Lincoln, NE, USA.) on sunny days from 9:00 to 11:00 a.m. in the early August 2016. The values for the following parameters were determined: net photosynthetic rate (P_n_), stomatal conductance (g_s_), intracellular CO_2_ concentration (C_i_), and transpiration rate (T_r_). Measurements were carried out with the following settings of the measuring equipment: 0.5 L min^−1^ air velocity, 25°C temperature, 70% relative humidity, 380 μmol mol^−1^ concentration of CO_2_ and 1,200 μmol m^−2^ s^−1^ PAR. Water use efficiency (WUE) was calculated as WUE = P_n_/T_r_, and instantaneous carboxylation efficiency (CUE) as CUE = P_n_/C_i_.

Fluorescence parameters were measured by PAM-2500 (WALZ Inc, Germany). Leaves were well dark-adapted, and then exposed to a weak actinic light (0.05 μmol m^−2^ s^−1^) to detect the initial minimal fluorescence (*F*_0_). Then a saturating light pulse (6,000 μmol m^−2^ s^−1^) was applied for 2 s to detect the maximal fluorescence (*F*_*m*_). The maximal potential quantum yield of photosystem PSII was calculated as *F*_*v*_/*F*_*m*_ = (*F*_*m*_−*F*_*o*_)/*F*_*m*_. Leaves were exposed to the saturating light pulse repeatedly in order to determine the non-photochemical quenching (qN) (Genty et al., 1989), and the effective quantum yield of PSII during actinic illumination (Φ_PSII_) was determined following Murchie and Lawson (2013). A detailed documentation of the measurement of the fluorescence parameters is given by Murchie and Lawson (2013). It is to be noted that *F*_v_/*F*_m_ and Φ_PSII_ reflect the maximal and effective quantum efficiency of PSII photochemistry, respectively, so that high values of these parameters indicate a good health status of the plant (Murchie and Lawson, 2013). Chlorophyll content was measured by portable chlorophyll meter SPAD-502 (Minolta Camera Co. Ltd., Osaka, Japan). For each leaf, the mean of five readings was used to determine the chlorophyll content.

### Laboratory measurements

After measuring the *in situ* parameters, the measured leaves were collected into plastic bags and immediately transported to the laboratory for laboratory measurements. Fresh samples of leaves (0.5 g) were ground in a tissue grinder, containing 5 ml of 50 mM potassium phosphate buffer (pH 7.8). After centrifugation at 15,000 g in 4°C for 15 min, the homogenate was stored in 4°C for analysis of soluble protein concentration and enzyme activities (SOD, POD, and CAT). Soluble protein content was determined following the guaiacol oxidation method described by Bradford (1976), using bovine serum albumin as the standard. The superoxide dismutase (SOD) activity was measured by the method of Giannopolitis and Ries (1977), where the activity is determined by the inhibiting effect of nitroblue tetrazolium (NBT) on the photoreduction. The peroxidase (POD) and catalase (CAT) activity was determined by the method of Chance and Maehly (1955). The reaction was conducted at 470 and 240 nm, and extinction coefficients of 2.47 Mm cm^−1^ and 36 m M cm^−1^ were used to determine the activity of POD and CAT, respectively.

In order to measure the foliar nutrient contents, the leaves were oven-dried, first in 105°C for 30 min, and then in 65°C to a constant weight, and then ground with a grinder (DFT-50A, Wenling LINDA Machinery Co. Ltd., China). The total content of carbon (C) and nitrogen N in the leaves was determined by a high-sensitivity CN analyzer Sumigraph NC-80 (Sumitomo Chemical Industry Co. Ltd., Japan). The phosphorus (P) content was determined by a modified Kjeldahl method followed by photometric analysis (Song et al., 2014).

### Statistical analyses

For the statistical analysis of the effect of N deposition and bamboo age on the physiological parameters addressed in this study, the data were subjected to a two-way ANOVA analysis. One-way ANOVA was used for analyzing the effect of N deposition on each parameter. *Post-hoc* one-way ANOVA was used to determine the pair-wise differences when a significant effect of bamboo age was found. Statistically significant differences were reported at *P* < 0.05. The association between net photosynthetic rate (P_n_) and foliar N concentration was determined by Pearson correlation analysis. All of the data were analyzed using SPSS statistical software (version 19.0, Armonk, New York, USA).

## Results

### Photosynthetic parameters

**Figure 1:**
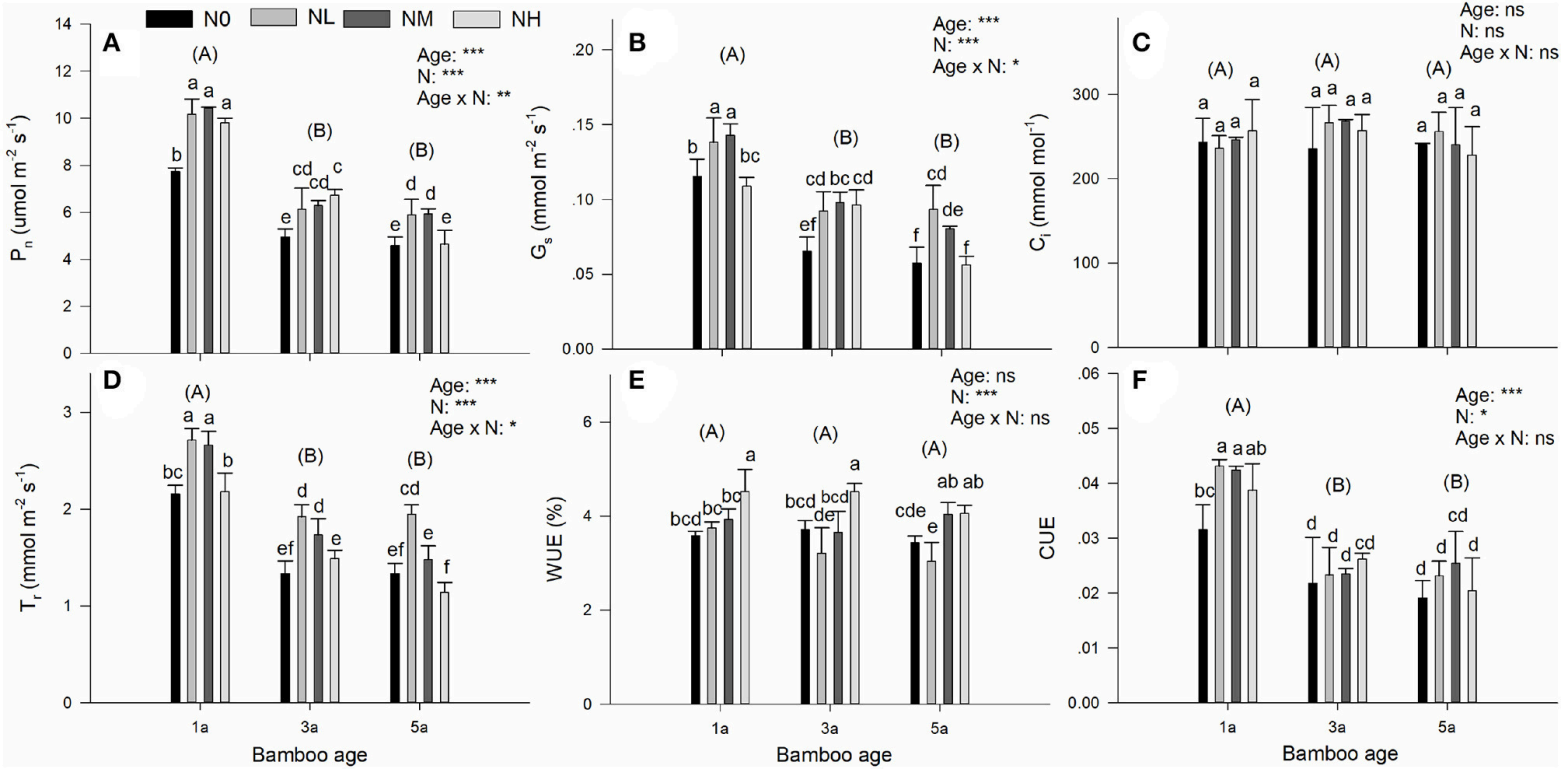
Effects of nitrogen deposition and tree age on gas exchange parameters in Moso bamboo leaves in a factorial experiment. One-year-old (1a), 3-year-old (3a), and 5-year-old (5a) trees were included in the experiment. Nitrogen deposition treatments: Ambient deposition with no addition (N0, control), low addition (NL = 30 kg N ha^−1^ year^−1^), medium addition (NM = 60 kg N ha^−1^ year^−1^), and high addition (NH = 90 kg N ha^−1^ year^−1^). Each treatment had three replicates (*n* = 3). **(A)** Net photosynthetic rate (P_n_), **(B)** stomatal conductance (g_s_), **(C)** intracellular CO_2_ concentration (C_i_), **(D)** transpiration rate (T_r_), **(E)** water use efficiency (WUE), and **(F)** instantaneous carboxylation efficiency (CUE). For each parameter mean ± SE is indicated. Bars with different lower-case letters indicate significant differences between all combinations of treatment and age, whereas groups of bars with different upper-case letters in parentheses indicate significant differences between the tree age classes (α = 0.05 in *post-hoc* one-way ANOVA combined with pair-wise Tukey's tests). A summary of the results of a two-way ANOVA addressing the effects of nitrogen deposition and tree age is seen in the upper right-hand corner of each panel (*0.01 ≤ *P* < 0.05; **0.001 ≤ *P* < 0.01; ^***^P < 0.001; ns, non-significant).

We found significant effects of bamboo age and nitrogen (N) deposition, and also significant interactions of them, on net photosynthetic rate (P_n_), stomatal conductance (g_s_), and transpiration rate (T_r_) (Figure 1). Compared with bamboos of the two older age classes, 1-year-old (1a) bamboos had significantly higher P_n_, g_s_, and T_r_ (Figures 1A,B,D). Compared with the treatments without N addition (N0), both the low (NL) and the medium (NM) additional N deposition increased P_n_, g_s_, and T_r_ in all age classes of bamboo (Figures 1A,B,D). However, no significant difference was found between the NL and NM treatments (Figures 1A,B,D), except that medium N addition significantly decreased T_r_ in comparison to low N addition in 5-year-old bamboos (Figure 1D).

Many decreasing trends occurred in responses to the high additional N deposition (NH). Accordingly, high N addition caused significant decreases of P_n_ and g_s_ in 5-year-old bamboos and a significant decrease of T_r_ in all age classes of the bamboos (Figures 1A,B,D). For intracellular CO_2_ concentration (C_i_), no significant main effect, or interaction, of age and N deposition was detected (Figure 1C). For water use efficiency (WUE), we found a significant effect of N deposition, but no significant age effect, or interaction of the two factors (Figure 1E). This significant effect on WUE was seen as a significant increase in WUE in response to high additional N deposition in all age classes of bamboos (Figure 1E). For instantaneous CUE, we found significant effects of both age and N deposition, but no significant interaction of them (Figure 1F). One-year-old bamboo showed higher CUE than bamboos belonging to the two older age classes (Figure 1F). A significant effect of N deposition on CUE was caused by the effect of N deposition on CUE in 1-year-old bamboos (Figure 1F).

### Chlorophyll and fluorescence parameters

**Figure 2:**
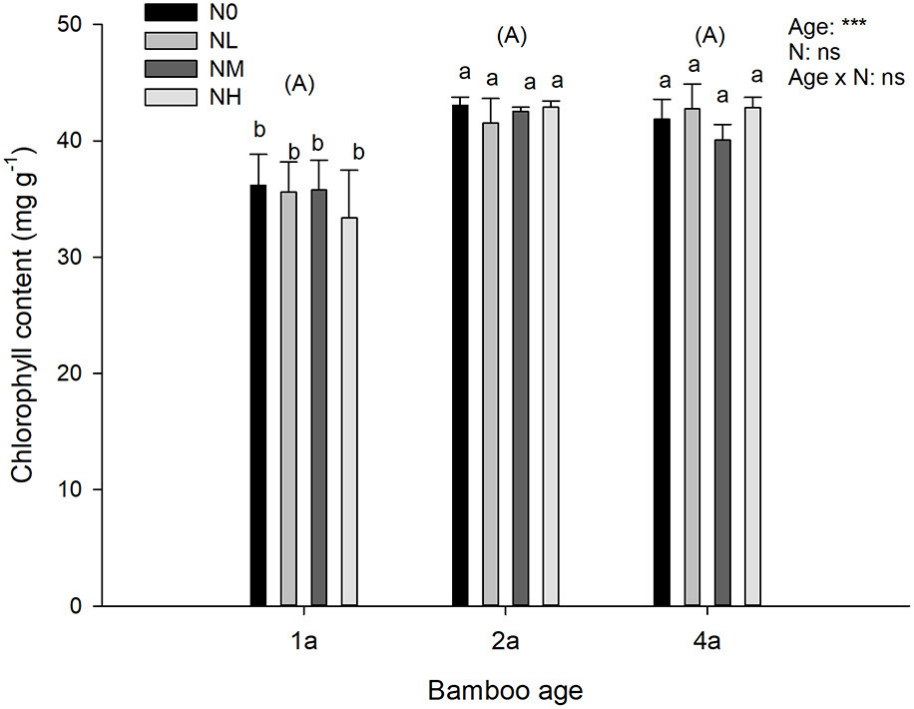
Effects of nitrogen deposition and tree age on chlorophyll content in Moso bamboo leaves in a factorial experiment. One-year-old (1a), 3-year-old (3a), and 5-year-old (5a) trees were included in the experiment. Nitrogen deposition treatments: Ambient deposition with no addition (N0, control), low addition (NL = 30 kg N ha^−1^ year^−1^), medium addition (NM = 60 kg N ha^−1^ year^−1^), and high addition (NH = 90 kg N ha^−1^ year^−1^). Each treatment had three replicates (*n* = 3). For the chlorophyll content mean ± SE is indicated. Bars with different lower-case letters indicate significant differences between all combinations of treatment and age, whereas groups of bars with different upper-case letters in parentheses indicate significant differences between the tree age classes (α = 0.05 in *post-hoc* one-way ANOVA combined with pair-wise Tukey's tests). A summary of the results of a two-way ANOVA addressing the effects of nitrogen deposition and tree age is seen in the upper right-hand corner of the panel (^***^P < 0.001; ns, non-significant).

For chlorophyll content, we found a significant effect of age, but no significant main N deposition effect, or significant interaction of the two factors (Figure 2). The significant effect of age was caused by an increase in chlorophyll content in response to the increasing age of bamboos (Figure 2).

**Figure 3:**
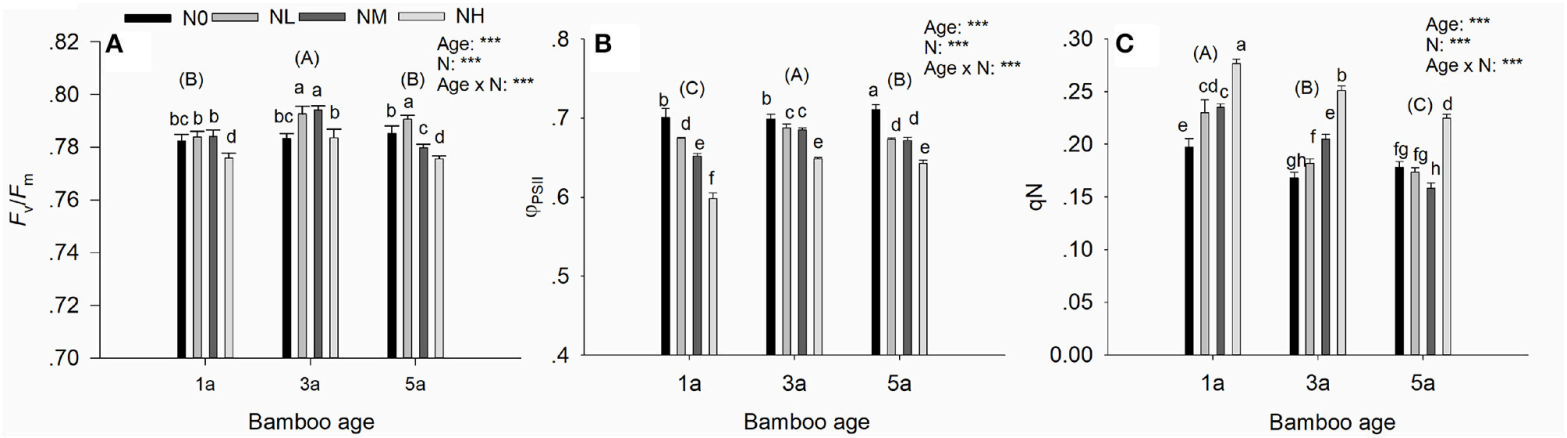
Effects of nitrogen deposition and tree age on fluorescence parameters in Moso bamboo leaves in a factorial experiment. One-year-old (1a), 3-year-old (3a), and 5-year-old (5a) trees were included in the experiment. Nitrogen deposition treatments: Ambient deposition with no addition (N0, control), low addition (NL = 30 kg N ha^−1^ year^−1^), medium addition (NM = 60 kg N ha^−1^ year^−1^), and high addition (NH = 90 kg N ha^−1^ year^−1^). Each treatment had three replicates (*n* = 3). **(A)** maximal quantum yield of photosystem II (*F*_v_/*F*_m_), **(B)** PSII operating efficiency (Φ_PSII_), and **(C)** non-photochemical quenching (qN). For each parameter mean ± SE is indicated. Bars with different lower-case letters indicate significant differences between all combinations of treatment and age, whereas groups of bars with different upper-case letters in parentheses indicate significant differences between the tree age classes (α = 0.05 in *post-hoc* one-way ANOVA combined with pair-wise Tukey's tests). A summary of the results of a two-way ANOVA addressing the effects of nitrogen deposition and tree age is seen in the upper right-hand corner of each panel (^***^P < 0.001; ns, non-significant).

For all fluorescence parameters *F*_v_/*F*_m_, Φ_PSII_, and qN, significant main effects of both age and N deposition were detected, and their interaction was also significant (Figures 3A–C). The significant effect of age was caused by a significant decrease in the non-photochemical quenching (qN) (Figure 3C). For the maximum potential (*F*_v_/*F*_m_) and the effective (Φ_PSII_) quantum yield of photosystem II, a humped age response was found as their values first increased from 1a to 3a, and then decreased at 5a (Figures 3A,B).

For the fluorescence parameters, a significant effect of N deposition and its significant interaction with age was seen in the following responses: With increasing N deposition, Φ_PSII_ decreased (Figure 3B) and qN increased (Figure 3C) in all age classes of Moso bamboo except that qN was significantly decreased by the medium additional N deposition for 5-year-old bamboo, as compared with the control treatment. Compared with the control, *F*_v_/*F*_m_ was increased in the low additional N deposition treatment NL, but contrary to the two older age classes, the increase was not significant in the 1-year-old bamboos (Figure 3A). A similar response was seen to the medium additional N deposition (NM) except that *F*_v_/*F*_m_ was significantly decreased in 5-year-old bamboos. The high additional N deposition caused a significant decrease in *F*_v_/*F*_m_ in all age classes of the bamboos (Figure 3A).

### Soluble protein contents and antioxidant enzyme activities

**Figure 4:**
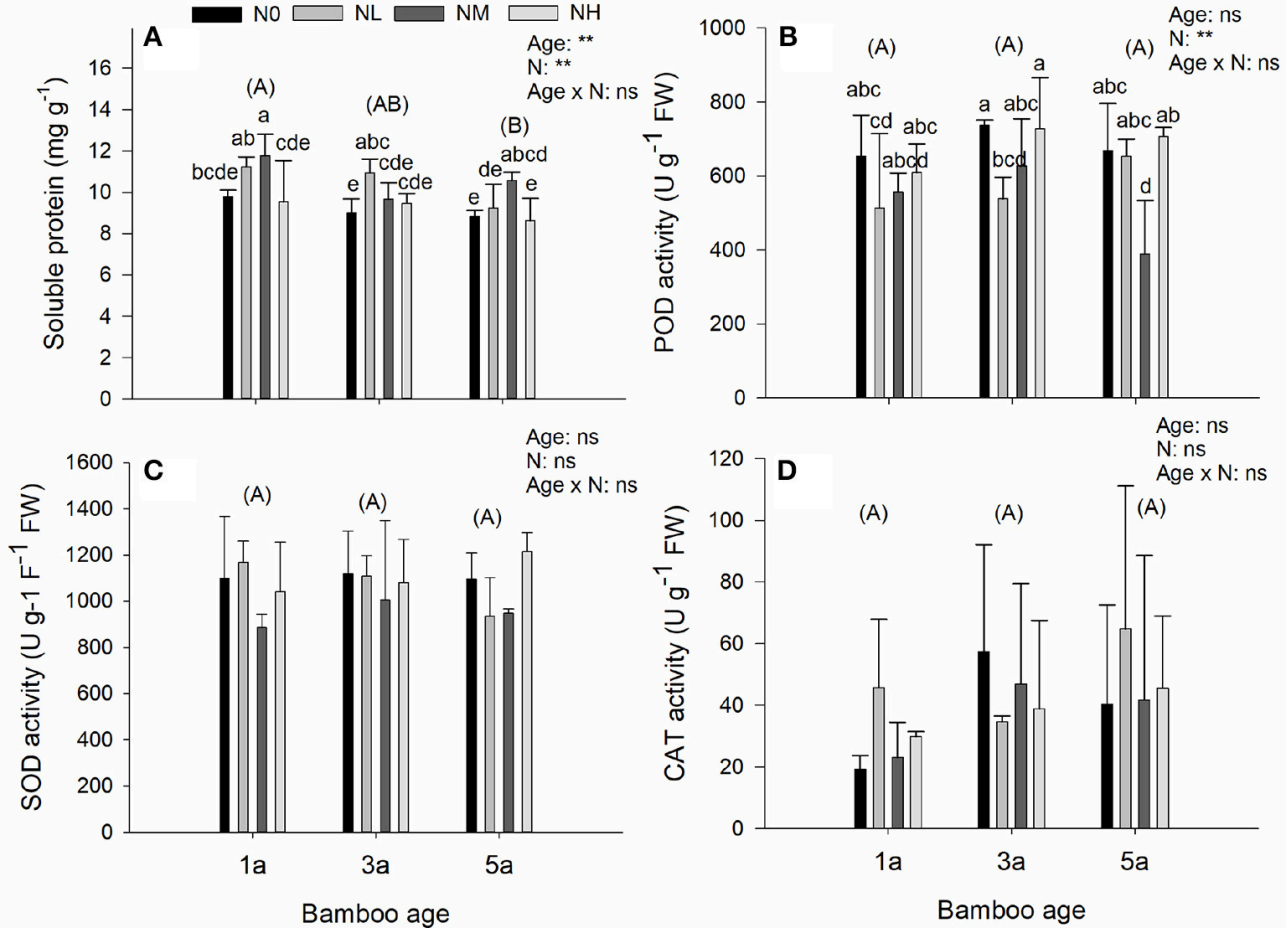
Effects of nitrogen deposition and tree age on soluble protein contents and antioxidant enzyme activities in Moso bamboo leaves in a factorial experiment. One-year-old (1a), 3-year-old (3a), and 5-year-old (5a) trees were included in the experiment. Nitrogen deposition treatments: Ambient deposition with no addition (N0, control), low addition (NL = 30 kg N ha^−1^ year^−1^), medium addition (NM = 60 kg N ha^−1^ year^−1^), and high addition (NH = 90 kg N ha^−1^ year^−1^). Each treatment had three replicates (*n* = 3). **(A)** Soluble protein content, **(B)** peroxidase activity (POD), **(C)** superoxide dismutase activity (SOD), and **(D)** catalase activity (CAT). For each parameter mean ± SE is indicated. Bars with different lower-case letters indicate significant differences between all combinations of treatment and age, whereas groups of bars with different upper-case letters in parentheses indicate significant differences between the tree age classes (α = 0.05 in *post-hoc* one-way ANOVA combined with pair-wise Tukey's tests). A summary of the results of a two-way ANOVA addressing the effects of nitrogen deposition and tree age is seen in the upper right-hand corner of each panel (^**^0.001 ≤ *P* < 0.01; ns, non-significant).

For the soluble protein and the enzyme parameters, we found a significant effect of age and of N deposition, but their interaction was not significant (Figure 4). Soluble protein content decreased with increasing age of bamboo (Figure 4A). The significant effect of N deposition was caused by an increase of soluble protein contents in response to low and medium additional N depositions, as compared with the control, and then a decrease in response to high additional N deposition (Figure 4A). For the antioxidant enzyme parameters we found no significant age or N deposition effect, or any significant interactions of them, except that POD was significantly affected by N deposition (Figures 4B–D). Compared with the control, POD decreased in response to low and medium additional N deposition, and then increased in response to high additional N deposition (Figure 4B).

### Foliar stoichiometry

**Figure 5:**
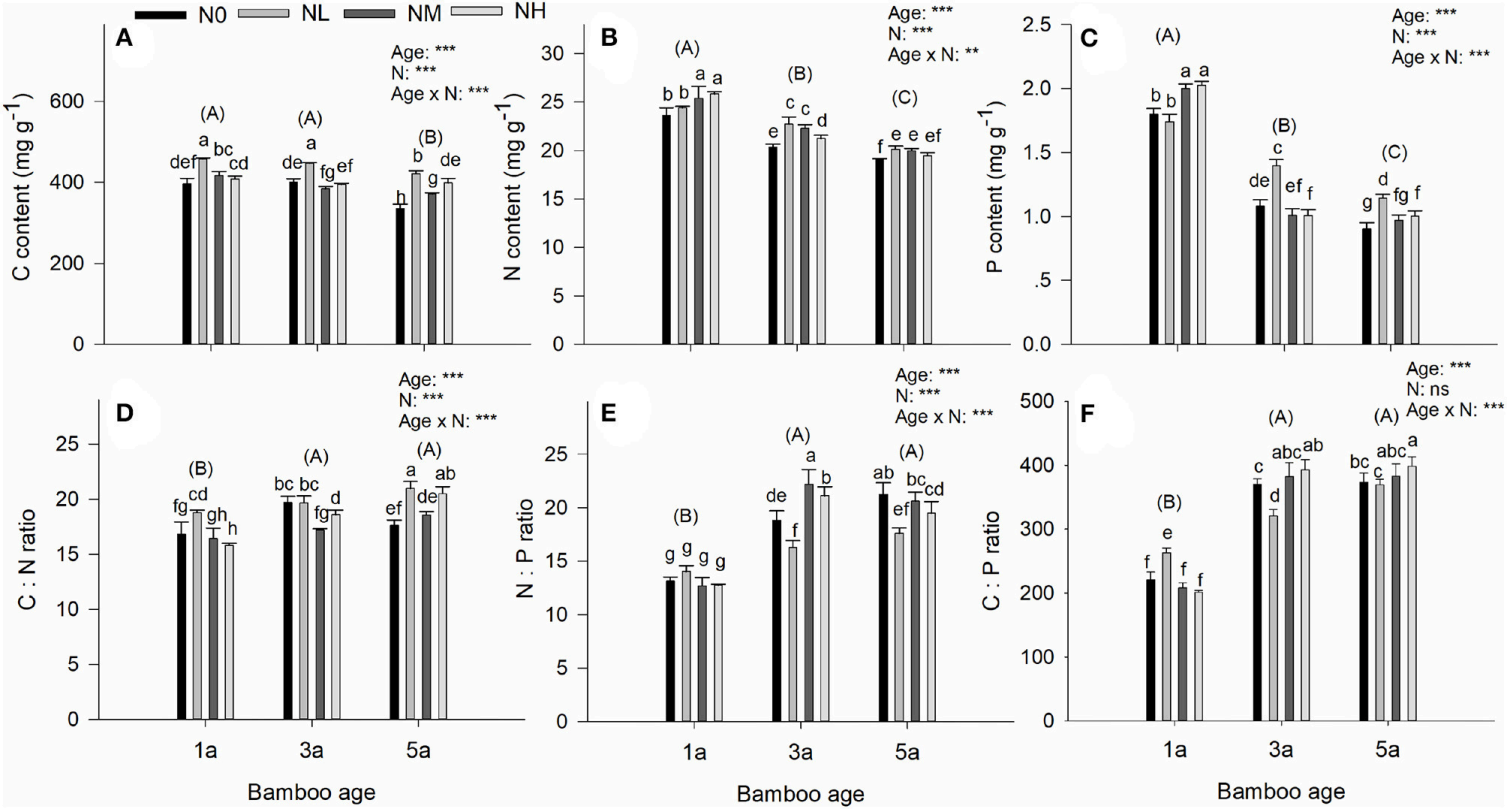
Effects of nitrogen deposition and tree age on foliar stoichiometry in Moso bamboo leaves in a factorial experiment. One-year-old (1a), 3-year-old (3a), and 5-year-old (5a) trees were included in the experiment. Nitrogen deposition treatments: Ambient deposition with no addition (N0, control), low addition (NL = 30 kg N ha^−1^ year^−1^), medium addition (NM = 60 kg N ha^−1^ year^−1^), and high addition (NH = 90 kg N ha^−1^ year^−1^). Each treatment had three replicates (*n* = 3). **(A)** Carbon content (C), **(B)** nitrogen content, (N), **(C)** phosphorus content (P), **(D)** C:N ratio, **(E)** N:P ratio, and **(F)** C:P ratio. For each parameter mean ± SE is indicated. Bars with different lower-case letters indicate significant differences between all combinations of treatment and age, whereas groups of bars with different upper-case letters in parentheses indicate significant differences between the tree age classes (α = 0.05 in *post-hoc* one-way ANOVA combined with pair-wise Tukey's tests). A summary of the results of a two-way ANOVA addressing the effects of nitrogen deposition and tree age is seen in the upper right-hand corner of each panel (^**^0.001 ≤ *P* < 0.01; ^***^P < 0.001; ns, non-significant).

For all of the foliar stoichiometric parameters (C, N, P, C/N, C/P, and N/P), significant effects of age, N deposition and their interaction were found, except that C/P was not affected by N deposition (Figure 5). The significant effects of age were caused by a significant decrease in C, N, and P (Figures 5A–C), and a significant increase in C/N, C/P, and N/P (Figures 5D–F), in response to the increasing age of the bamboos. For C, N, and P, the main effects of N deposition and its interaction with age were not completely similar with each other (Figures 5A–C). For C content (Figure 5A), all age classes of bamboos showed higher C in the treatment with low additional N deposition than in the control. However, with medium and high additional N depositions, differences were found among the age classes. For N content (Figure 5B), all the additional N deposition treatments caused N content to increase relative to the control in all age classes of bamboo; but the highest N content occurred at high additional N deposition treatment in 1-year-old bamboo and at low N deposition treatment in the two older age classes. Compared to the low additional N deposition treatment, increasing N addition caused an increase in the P content in 1-year-old bamboo, but a decrease in the two older age classes (Figure 5C). For C/P, N/P, and C/P, the responses to N deposition in the various age-classes were diverse, showing no constant patterns (Figures 5D–F).

## Discussion

### Photosynthetic activity, foliar chemistry, and their relationships

Our aim was to evaluate the effects of additional N deposition on physiological responses in Moso bamboo plantations in subtropical conditions. This information is relevant both for understanding how globally increasing atmospheric N deposition may affect performance of bamboo of various ages and for assessing the potential risks caused by high N load to the subtropical forest.

Our results suggest that N deposition affects net photosynthetic rate (P_n_), stomatal conductance (g_s_), and transpiration rate (T_r_) in Moso bamboo. Furthermore, the responses changed with the age of the trees, indicating an interaction of the N deposition and age. For all ages of bamboo, P_n_, g_s_, and T_r_, were increased by low additional N deposition. However, increasing N addition generally did not bring additional increases in the values of these parameters. Peculiarly, in the case of the 5-year-old bamboo, high additional N deposition significantly suppressed P_n_ and g_s_. Similarly, Granath et al. (2009b) found that P_n_ started to decrease at high N load in *S. balticum*. These results support our first hypothesis that photosynthetic activity in bamboo is sensitive to N deposition with low N addition increasing the rate of net photosynthesis and further N addition causing no further change in it. The results also show, for the first time, that P_n_ in 5-year-old bamboo is less tolerant to extremely high N deposition than the P_n_ in younger bamboo. The different response of the three age classes of bamboo can be explained by different requirements of N in different bamboo growth stages (Kursar and Coley, 2003). Accordingly, we inferred that leaves of the 5-year-old bamboo required less N than those of the fast-expanding younger ones.

To examine our second hypothesis of changes in photosynthetic activity being associated with foliar chemistry, we further investigated the responses of foliar chemistry to N deposition. We found that in both 3- and 5-year-old bamboo, foliar N was significantly increased under low additional N deposition. This is consistent with similar responses found earlier in many other plants (Arróniz-Crespo et al., 2008; Granath et al., 2009b). However, further increased additional N deposition did not cause increased N concentration in the leaves of 3- and 5-year-old bamboo, indicating that they were readily N saturated under low additional N deposition. By contrast, 1-year-old bamboo seemed to require more N, since N saturation was not reached until under medium additional N deposition. Overall, moderate additional N deposition increased foliar N up to a threshold level, while further increased N deposition brought no additional increase in N concentration in the bamboo leaves.

Furthermore, we found that photosynthetic rate (P_n_) was highly correlated with foliar N concentration (Figure S1), indicating that the bamboo trees were able to take advantage of the increased availability of the N nutrient. A similar linear relationship between P_n_ and tissue N has been also reported in other vascular plants (Reich et al., 1999; Hikosaka, 2004). Compared with some bog shrubs (i.e., *Chamaedaphne calyculata, Vaccinium myrtilloides*) with no change in photosynthetic capacity in response to the increased tissue N (Bubier et al., 2011), Moso bamboo can better acclimate to an environment of high concentration of N.

Foliar stoichiometry is a useful tool to assess nutrient limitation (Elser et al., 2009; Blanes et al., 2013). In this study, we found C/N/P stoichiometric ratios were very sensitive to N deposition in all three ages of bamboo. However, there were differences among the age classes of bamboo in their responses of the C/N/P ratios to the N deposition. In particular, high additional N deposition resulted in no change, an increase, and a decrease of the N/P ratio in 1-, 3-, and 5-year-old bamboo, respectively. This indicated that Moso bamboos of various age-classes might have different regulatory mechanism of nutrition allocation during acclimation to high N load stress. These results suggest that foliar stoichiometric ratios may not be generally reliable indicators of N deposition impacts in bamboo.

### Potential stress induced by extremely high N deposition

Granath et al. (2009a) found weak relationships between P_n_ (or foliar N content) and N load in *Sphagnum* species. In line with their findings, our results suggested, for the first time for a tall vascular plant, that P_n_ (or foliar N) may not be a useful indicator of critically high N load causing harmful impacts in bamboo, since increased N deposition first increased P_n_ (or foliar N) up to a threshold level, then with successively higher N loads the effect saturated, and no direct toxic effect of N on P_n_ (or foliar N) was found in any of the three bamboo age classes. The only exception was that P_n_ was significantly suppressed by high additional N deposition in 5-year-old bamboo. This raised our concern that there is a critical N load beyond which further N load may lead to potential plant stress. Actually, in some bryophytes the biomass accumulation can decrease even though P_n_ is not decreased by N load (Granath et al., 2009b).

Stomatal conductance and transpiration rate may give early indications of shifts in physiology of plants that occur with N addition (Ward et al., 2015). In support of our third hypothesis, we found that a drought stress was potentially induced by high N additional deposition, as indicated by the suppressed g_s_ and T_r_ (Flexas et al., 1999) in response to the high additional N deposition, especially in 5-year-old bamboo. This is consistent with findings in *Pinus taeda* where long-term sensitivity to drought was induced as shown by the transpiration reduction triggered by N addition (Ward et al., 2015). Interestingly, we found WUE was enhanced by high N addition, probably due to the higher availability of the N nutrient. Increased WUE is often related to enhanced allocation of C to shoot rather than root growth, which may make plants more vulnerable to drought stress (Graciano et al., 2005). Similarly, N deposition has been found to increase susceptibility to drought in *Molinia caerulea* (Friedrich et al., 2012) and *Artemisia californica* (Valliere and Allen, 2016); and in both of these species the increased drought susceptibility was caused by the decreased allocation to roots caused by N addition.

Given that chlorophyll fluorescence reflects photosynthetic activities and can give insight into the stress tolerance in plants (Maxwell and Johnson, 2000; Souza et al., 2004), we further investigated whether the photochemical response to N deposition can detect the potential stress induced by N deposition in bamboo. Our results show that *F*_v_/*F*_m_ is generally increased by low additional N deposition; while high additional N deposition resulted in a suppressed *F*_v_/*F*_m_ in all ages of bamboo, indicating reduced potential photosynthetic rates (Demmig-Adams and Adams, 1996). Suppressed *F*_v_/*F*_m_ may be a result of environmental stresses, such as water stress (Tremblay et al., 2012). Thus, in our study the supressed *F*_v_/*F*_m_ ratios may have been caused by water stress induced by the additional N deposition. This explanation is supported by the suppressed T_r_ and increased WUE under high additional N deposition. Furthermore, *F*_v_/*F*_m_ can be broadly used as an indicator of critically high N load for all ages of bamboos since *F*_v_/*F*_m_ was suppressed by high N addition in all age classes of bamboo, though moderate N addition increased it (Figure 3C).

Moreover, in our study, Φ_PSII_ was negatively affected by N deposition. Additional N deposition resulted in a significantly decreased Φ_PSII_ in comparison to control treatment, indicating reduced actual photosynthetic rates (Demmig-Adams and Adams, 1996). These findings are consistent with similar responses seen in bryophytes where effective quantum yield of PSII decreased under increased N deposition (Arróniz-Crespo et al., 2008). In addition, Φ_PSII_ can be used as indicator to monitor N deposition rate in the bamboo forest, since Φ_PSII_ was generally decreased as the N deposition rate increased (Figure 3B). Furthermore, in our study increased N deposition generally contributed to the increased qN, indicating diminished photo-protection capacity and increased risk of the photo-damage (Demmig-Adams and Adams, 1996). Overall, while *F*_v_/*F*_m_ has been previously served as an indicator of N deposition (Arróniz-Crespo et al., 2008), our study suggested that Φ_PSII_ and qN are also very sensitive to N deposition and should be used as additional useful indicators of N deposition impacts in bamboo. In particular, *F*_v_/*F*_m_ can be used as a good indicator of critically high N load, while Φ_PSII_ can be used to monitor N deposition rates. Notably, stress in bamboo induced by high N deposition can be detected by suppressed *F*_v_/*F*_m_, appearing with reduced Φ_PSII_, and increased qN.

Inconsistent with the earlier findings that increased N application increases the chlorophyll content (Granath et al., 2009b; Yao et al., 2016; Zhang et al., 2016), we found that chlorophyll content was not significantly affected by N deposition in Moso bamboo. A similar lack of response was also observed in *R. squarrosus* (Arróniz-Crespo et al., 2008). Thus, impacts of N on chlorophyll seemed to be species specific, and chlorophyll content may be not an appropriate indicator of N deposition impacts on bamboo. However, further measurements of pigment compositions of Chl *a/b* and carotenoids are suggested to comprehensively reveal the impacts of N addition on photosynthesis related parameters (Arróniz-Crespo et al., 2008).

Soluble protein contents showed sensitivity in response to N deposition in bamboo. In all the three age classes of bamboo, the concentration of soluble proteins was generally increased by medium additional N deposition (Figure 4A). This response was probably due to N being as an essential structural component of proteins (Rees et al., 2005). Accordingly, the deposited N was directly absorbed by the bamboos and readily transformed into protein. However, high additional N deposition caused a decrease in soluble proteins, indicating that leaf metabolic functions may be impaired as a result of excess N deposition (Guy, 1990). A similar pattern of change in soluble proteins was also found in *Syntrichia caninervis* (Zhang et al., 2016).

Abiotic stress by excess of certain nutrients can cause increases in antioxidant enzyme activities (Fang et al., 2002; Mittler, 2002; Medici et al., 2004). However, the antioxidant system can also be up-regulated in order to protect from photo-oxidative damage caused by N limitation (Logan et al., 1999; Chen and Cheng, 2003). Among the antioxidant enzyme parameters, peroxidase (POD) has been found to be more sensitive to N deposition than superoxide dismutase (SOD) and catalase (CAT) in *Sytrichia caniervis* (Zhang et al., 2016), whereas in *Eugenia uniflora* all three enzymes have been found to be sensitive to N deposition (Neves et al., 2009).

In our study N deposition significantly affected POD, but not SOD or CAT activity. In comparison to the control treatment, the low and medium N additions alleviated N deficiency causing a decrease in POD activity. However, high additional N deposition caused an increase in POD activity, indicating negative effects on bamboo growth and physiology. A similar pattern of change in POD activity in response to N deposition was also found in a xerophytic moss (Zhang et al., 2016) and in a restinga shrub (Neves et al., 2009). Overall, our results suggested, for the first time, that both soluble protein contents and POD activity are sensitive to N deposition in bamboo, so that they should be valuable indicators to monitor the stress induced by N deposition in subtropical bamboo trees.

## Conclusions

This study provides a novel comprehensive evaluation of multiple physiological responses to N deposition impacts in three age classes of Moso bamboo. This is needed to assess the N deposition threat to vascular plants without visible symptoms of injury. We found that photosynthetic rate highly correlated with foliar nitrogen concentration, increasing under all levels of additional nitrogen deposition in the range of from 30 to 90 kg N ha^−1^ year^−1^. However, P_n_ or foliar N may not be a robust indictor of N deposition impacts, since both of them had a saturating response to the additional N deposition. Furthermore, the highest applied additional N deposition (90 kg N ha^−1^ year^−1^) may induce physiological stress to Moso bamboo. This was suggested by the reduced potential and actual photosynthetic use of light energy, diminished photo-protection capacity, increased risk of the photo-damage, decrease in soluble protein contents, and the increase in POD activity. These responses can be detected by sensitive indicators, including *F*_v_/*F*_m_, subsequently corroborated with Φ_PSII_, qN, foliar soluble protein contents, and POD activity. Overall, our study suggested, for the first time, that though photosynthetic rate and foliar N increased in response to increasing N deposition, changes in many physiological parameters indicated a negative effect in Moso bamboo. Thus, for the fast-growing vascular plants, responses to N deposition cannot be assessed simply from photosynthetic traits related to growth rate without considering the risk of susceptibility to stress induced by high N deposition revealed by other physiological parameters.

## Author contributions

XS, YY, RZ, and JW designed the experiments. QL, CP, and HY conducted the field and laboratory experiments. The manuscript was written by RZ and was revised by HH.

### Conflict of interest statement

The authors declare that the research was conducted in the absence of any commercial or financial relationships that could be construed as a potential conflict of interest.
