# Supplementary material for: Nitrogen Deposition Enhances Photosynthesis in Moso Bamboo but Increases Susceptibility to Other Stress Factors
Source: Front Plant Sci. 2017 Nov 16;8:1975. doi: 10.3389/fpls.2017.01975 (PMC5696719; doi:10.3389/fpls.2017.01975)

<u>**Fig. S1**</u>

Correlation between net photosynthetic rate (P_n) and foliar nitrogen content in Moso bamboo leaves in a factorial experiment. The association between P_n and foliar N concent was determined by Pearson correlation analysis.

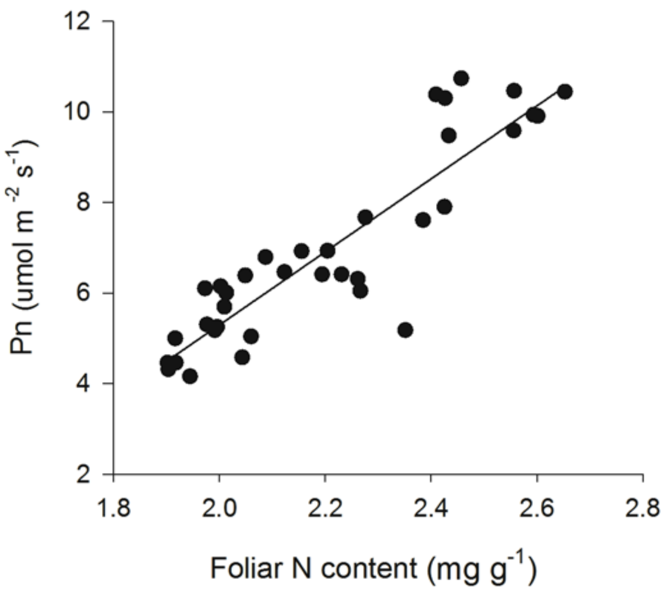

Supplement: Supplementary file 1 [file Image1.pdf]
